# Death of Dr. Jacobson, of Copenhagen

**Published:** 1843-10-28

**Authors:** 


### DEATH OF DR. JACOBSON, OF COPENHAGEN.

Dr. Louis Levi Jacobson, chief physician to their Majesties the King and Queen of Denmark, died at Copenhagen on the morning of the 29th of August last, after a short illness, aged sixty-one years. Dr. Jacobson was born in Copenhagen in 1782, of respectable Jewish parents. The numerous and learned works which he published, especially on anatomy, and which have been translated into all the European languages, led him to be appointed to two chairs—one in the University, and the other in the Academy of Surgery in Copenhagen—in spite of an ancient custom prevailing in Denmark, which excludes from the profession of teaching all others except those of the religion of the state, viz., that of the Confession of Augsburg. The present King of Denmark raised Dr. J. to the rank of Counseller of State; he was Chevalier of several orders, and was a member of almost all the learned societies of Europe. The Royal Academy of Sciences of Paris nominated him, in June, 1833, a corresponding member, in the place of the late Sir Everard Home; and, in the month of November of the same year, this learned body awarded to him the golden medal, of the value of 4,000 francs, for the invention of the *Lithoclastre*, or *Brise-Pierre*, an instrument for crushing calculi.

